# The interplay of StyR and IHF regulates substrate-dependent induction and carbon catabolite repression of styrene catabolism genes in *Pseudomonas fluorescens *ST

**DOI:** 10.1186/1471-2180-8-92

**Published:** 2008-06-11

**Authors:** Giordano Rampioni, Livia Leoni, Biancamaria Pietrangeli, Elisabetta Zennaro

**Affiliations:** 1 Department of Biology, University Roma Tre, Viale Marconi 446, 00146, Rome, Italy; 2 Istituto Superiore per la Prevenzione e la Sicurezza sul Lavoro, Dipartimento Insediamenti Produttivi ed Interazioni con l'Ambiente, Via Urbana 167, 00184, Rome, Italy

## Abstract

**Background:**

In *Pseudomonas fluorescens *ST, the promoter of the styrene catabolic operon, P*styA*, is induced by styrene and is subject to catabolite repression. P*styA *regulation relies on the StyS/StyR two-component system and on the IHF global regulator. The phosphorylated response regulator StyR (StyR-P) activates P*styA *in inducing conditions when it binds to the high-affinity site STY2, located about -40 bp from the transcription start point. A *cis*-acting element upstream of STY2, named URE, contains a low-affinity StyR-P binding site (STY1), overlapping the IHF binding site. Deletion of the URE led to a decrease of promoter activity in inducing conditions and to a partial release of catabolite repression. This study was undertaken to assess the relative role played by IHF and StyR-P on the URE, and to clarify if P*styA *catabolite repression could rely on the interplay of these regulators.

**Results:**

StyR-P and IHF compete for binding to the URE region. P*styA *full activity in inducing conditions is achieved when StyR-P and IHF bind to site STY2 and to the URE, respectively. Under catabolite repression conditions, StyR-P binds the STY1 site, replacing IHF at the URE region. StyR-P bound to both STY1 and STY2 sites oligomerizes, likely promoting the formation of a DNA loop that closes the promoter in a repressed conformation. We found that StyR and IHF protein levels did not change in catabolite repression conditions, implying that P*styA *repression is achieved through an increase in the StyR-P/StyR ratio.

**Conclusion:**

We propose a model according to which the activity of the P*styA *promoter is determined by conformational changes. An open conformation is operative in inducing conditions when StyR-P is bound to STY2 site and IHF to the URE. Under catabolite repression conditions StyR-P cellular levels would increase, displacing IHF from the URE and closing the promoter in a repressed conformation. The balance between the open and the closed promoter conformation would determine a fine modulation of the promoter activity. Since StyR and IHF protein levels do not vary in the different conditions, the key-factor regulating P*styA *catabolite repression is likely the kinase activity of the StyR-cognate sensor protein StyS.

## Background

Styrene is a basic building block for the manufacture of a broad range of products containing molecules such as polystyrene, butadiene-styrene latex, styrene copolymers and unsaturated polyester resins. These products range from packaging materials to food service items to a myriad of consumer electronics, construction, transportation and medical applications. Styrene exposure may cause contact-based skin inflammation, irritation of eyes, nose and respiratory tract, while neurological effects, such as alterations in vision, hearing loss and longer reaction times, have been associated with styrene exposure in the workplace [1]. Microbial biodegradation and polluted air biofiltration are attractive options for the removal of styrene from the environment, because they are cost-effective and do not generate secondary contaminants. Therefore styrene-degrading microorganisms have been receiving increasing interest, mainly concerning the factors that can help or impair the degradation process [2].

**Figure 1:**
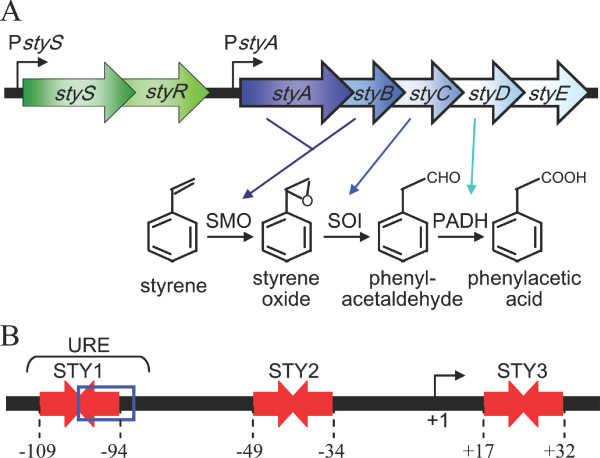
**The styrene-catabolic system of *P. fluorescens *ST**. (A) Organization of the *stySR *regulatory and of the *styABCDE *catabolic operons in *Pseudomonas *spp. *styS*, sensor histidine kinase; *styR*, response regulator; *styA *and *styB*, styrene monooxygenase (SMO); *styC*, styrene oxide isomerase (SOI); *styD*, phenylacetaldehyde dehydrogenase (PADH); *styE*, styrene transport protein. P*styS *and P*styA *are the promoters of the regulatory and catabolic operons, respectively. (B) Schematic representation of the P*styA *promoter region. Inverted red arrows, StyR-P-binding sites STY1, STY2 and STY3 (numbering refers to distance from P*styA *transcription start site). Blue box, IHF-binding site. Bent arrow, transcription start site.

Despite the large number of bacteria isolated for their capability to grow on styrene, genetic studies have essentially been performed on strains belonging to the genus *Pseudomonas *[2]. In these strains styrene degradation starts with the oxidation of the vinyl double bond to styrene oxide by styrene monooxygenase (SMO), a two-component flavin-dependent oxygenase encoded by the *styA *and *styB *genes, whose reaction mechanism has been proposed [3,4]. *styC *codes for styrene oxide isomerase (SOI), which converts styrene oxide to phenylacetaldehyde that is in turn oxidized to phenylacetic acid by phenylacetaldehyde dehydrogenase (PADH), encoded by *styD *[5-10]. The *styE *gene codes for a protein likely involved in the active transport of styrene [11]. These genes form an operon, named *styABCDE*, that is highly conserved in all the styrene-degrading *Pseudomonas *strains studied up to now (Figure 1A) [12]. Phenylacetic acid is a common substrate for *Pseudomonas *spp., probably because, beside styrene, degradation of many other aromatic compounds converge towards the formation of this compound [13].

In *Pseudomonas fluorescens *ST, as well as in the other styrene-degrading *Pseudomonas *strains, the expression of the *styABCDE *operon is regulated by the StyS/StyR two-component system, encoded by the *stySR *operon (Figure 1A) [9,10]. The product of the *styS *gene, StyS, is a protein of about 109 kDa, predicted to be a hybrid histidine kinase (HK) with several distinct functional domains. A distinctive feature of this protein with respect to other hybrid HKs is the presence of two different kinase cores (HK/ATPase domains). Moreover it contains an internal receiver domain and two putative input domains, consisting of the PAS and PAC sensory sub-domains [12]. The *styR *gene encodes a protein, StyR, of about 23 kDa disclosing the typical structural features of other response regulators [14]. Activation of StyR requires phosphorylation that triggers StyR dimerization and DNA binding [15].

Three distinct binding sites, endowed with different affinities for phosphorylated StyR (StyR-P), have been characterized on P*styA*, the promoter of the styrene catabolic operon (Figure 1B) [16]. Site STY2 is the highest-affinity StyR-P binding site. The DNA region upstream of STY2, named URE (Upstream Regulatory Element), contains a lower affinity StyR-P binding site (STY1) overlapping a binding site for the Integration Host Factor (IHF) global regulator, placed in the opposite helix with respect to STY1 site. However, when a promoter fragment containing both STY1 and STY2 was used in DNase I protection experiments, we found that StyR-P bound these sites simultaneously, indicating that StyR-P binding to these sites is cooperative. The lowest affinity StyR-P binding site (STY3) is located downstream of the transcription start point [16].

P*styA *activity is induced by styrene and partially repressed by the addition of glucose (60% repression) or of other more favourable carbon sources [17]. This phenomenon, named carbon catabolite repression, is frequently observed in the regulation of aromatic catabolic pathways in pseudomonads, although, in most cases, little is known about the mediators or the mechanisms underlying repression. In their whole, the studies carried out up to date rule out in *Pseudomonas *the involvement of cyclic AMP and indicates that different genes are involved in the catabolite repression of different catabolic pathways [reviewed in [18,19]].

Studies on the activity of deleted and/or mutated variants of P*styA*, in cells grown in inducing (growth on styrene as sole carbon source) or catabolite repression (glucose added to a styrene growing culture) conditions, showed that StyR-P acts as an activator when it binds to site STY2, and that this binding is essential for promoter activity [20]. Conversely, deletion of the URE decreased P*styA *activity and partially relieved it from glucose-mediated repression. When in this deleted promoter the STY3 site was also inactivated, glucose-mediated repression was completely abolished. From these data we concluded that StyR-P acts as a repressor when it binds to site STY3, and that the URE region of P*styA *is involved in both styrene-dependent induction and catabolite repression [16]. However, the relative role played by IHF and StyR-P on this *cis*-acting element remained unclear.

This study was undertaken to address this issue and to understand if changes in the relative levels of StyR-P and IHF could determine P*styA *catabolite repression.

The results obtained make it possible to propose a model according to which the fine regulation of the P*styA *promoter, in the different growth conditions, mainly depends on the phosphorylation levels of StyR, thus on the activity of the cognate StyS sensor kinase.

## Results and discussion

### StyR-P and IHF compete for binding to the URE region

The promoter region of the *styABCDE *operon is shown in Figure 1B. The P*styA *region located upstream of the STY2 site, referred to as URE (Upstream Regulatory Element), contains two *cis*-acting regulatory elements: an IHF binding site and the StyR-P binding site STY1 [16]. Since the STY1 and IHF binding sites overlap, it was postulated that StyR-P and IHF could compete for binding to the URE region. To clarify this issue, competition experiments were performed by DNase I protection assays on a DNA-probe encompassing site STY2 and the URE region of P*styA*.

**Figure 2:**
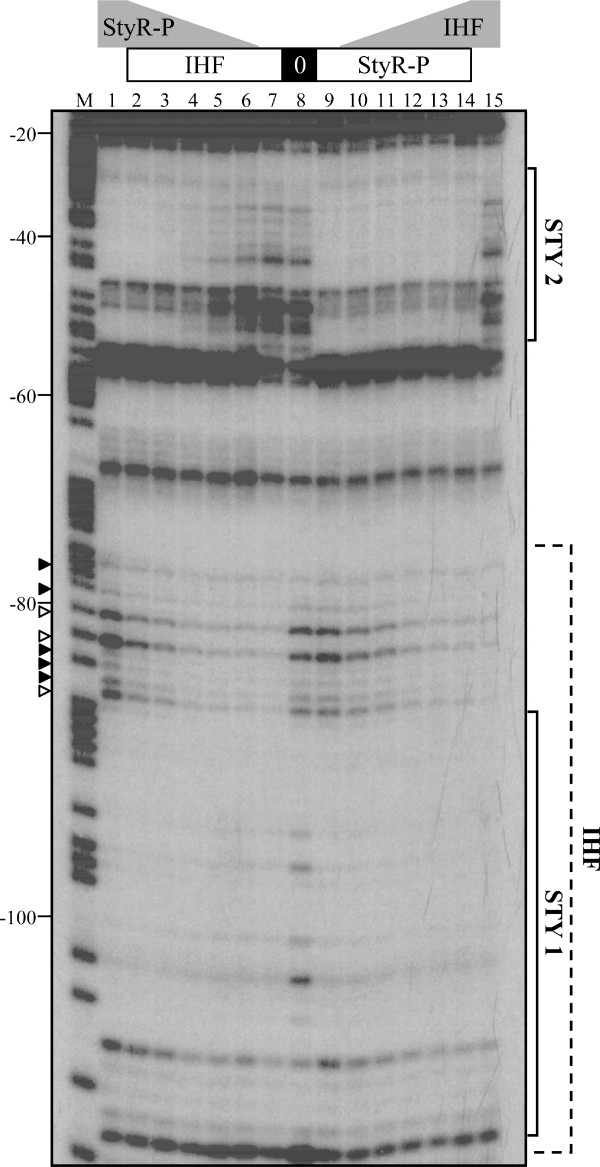
**Characterization of StyR-P and IHF binding to the URE**. DNase I protection assay in which a DNA fragment extending from -145 to -8 (Top Strand) of the P*styA *promoter was incubated with different amounts of IHF and/or StyR-P proteins prior to DNase I digestion. Numbering refers to P*styA *transcription start site. Solid brackets indicate the regions showing specific protection by StyR-P; dashed bracket indicates the region showing specific protection by IHF. Triangles indicate bands that are distinctive of the protection pattern obtained in the absence of IHF (open triangles indicate hypersensitive sites). M, Maxam and Gilbert sequencing reactions (A+G); IHF concentrations: lanes 1, 8, and 9, no IHF added; lanes 2 to 7, 4.0 μM; lanes 10 to 15, 0.5 μM, 1.0 μM, 2.0 μM, 4.0 μM, 8.0 μM and 8.0 μM, respectively. StyR-P concentrations: lanes 7, 8, and 15, no StyR-P added; lanes 1 to 6, 8.0 μM, 8.0 μM, 4.0 μM, 2.0 μM, 1.0 μM and 0.5 μM, respectively; lanes 9 to 14, 4.0 μM.

As shown in Figure 2, the IHF protection pattern (nucleotides -110/-75 from the P*styA *transcription start site; Figure 2, lanes 7 and 15) is wider than the StyR-P protection pattern on STY1 (nucleotides -110/-90 from the transcription start site; Figure 2, lanes 1 and 9), so that they are easily distinguishable. The addition of increasing StyR-P concentrations (Figure 2, lanes 6 to 2) to the IHF/probe preformed complex, led to the protection of site STY2, and to the replacement of the IHF protection pattern with the narrower StyR-P protection pattern on the URE region. In Figure 2 triangles indicate the bands that are distinctive of the pattern obtained in the absence of IHF. Three of these bands become hypersensitive sites under increasing concentrations of StyR-P (indicated by open triangles in Figure 2), suggesting a conformational change of P*styA *structure (see below). Likewise, the addition of increasing IHF concentrations (Figure 2, lanes 10 to 14) to the StyR-P/probe preformed complex, led to the replacement of the StyR-P protection pattern at the STY1 site with the wider IHF protection pattern. In this case the binding of IHF to the URE region did not affect the binding of StyR-P to site STY2.

In the whole, these data demonstrate that IHF and StyR-P are in binding competition for the URE region, and that IHF can bind the URE region without altering StyR-P binding to site STY2.

### StyR-P and IHF play opposite roles when bound to the URE region

**Figure 3:**
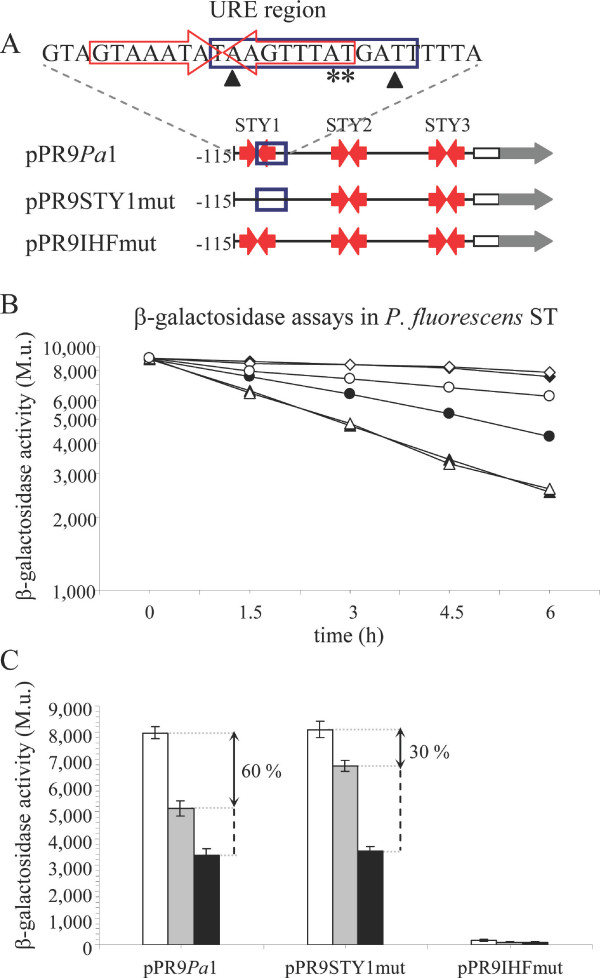
**Functional role of the URE region**. (A) Schematic representation of P*styA *and its mutated derivatives cloned in the promoter probe vector pPR9TT. Plasmid designations are given on the left. Red inverted arrows indicate the StyR-P binding sites. The IHF binding site is blue-boxed. Nucleotides numbering is referred to the P*styA *transcription start site. The white rectangle indicates the *styA *ORF. The grey arrow indicates the *lacZ *gene fused to *styA*. A detail of the URE region is reported on the top. Asterisks and triangles indicate nucleotides mutated (A→T and T→A substitutions) to generate the pPR9STY1mut and pPR9IHFmut constructs, respectively. (B) β-galactosidase activities disclosed by *P. fluorescens *ST strains carrying the different pPR9TT-derivative plasmids represented in (A). Filled symbols, pPR9*Pa*1; open symbols, pPR9STY1mut; diamonds, cultures growing on styrene; circles, styrene-grown precultures to which both styrene and 0.4% glucose were added at time zero; triangles, styrene-grown precultures to which only 0.4% glucose was added at time zero. (C) β-galactosidase activities measured after two exponential cell divisions on styrene (white bars), on styrene plus 0.4% (wt/vol) glucose (grey bars), or on 0.4% (wt/vol) glucose (black bars), are reported in the histogram. The extent of glucose mediated repression in carbon catabolite repression condition is indicated by the double arrow line. Repression was calculated by assuming that the promoter activity in styrene was 100% and the promoter activity in glucose corresponded to 100% repression. Standard deviations (vertical lines) are based on the mean values of five independent experiments.

In a previous work we showed that deletion of the URE led to a decreased promoter activity in inducing conditions (growth on styrene as sole carbon source) and to a partial desensitization to carbon catabolite repression (growth on styrene plus glucose). Actually, P*styA *activity was 60% reduced in catabolite repression, while it was only 38% reduced in the URE-deleted variant of the promoter in the same growth conditions [16]. To better understand the contribution of StyR-P and IHF in determining the P*styA *activity under styrene induction and glucose catabolite repression, we generated two variants of this promoter: *Pa1*STY1mut and *Pa1*IHFmut, each containing nucleotide substitutions such that impair StyR-P binding to site STY1 or IHF binding to the URE, respectively (Figure 3A). Electrophoretic mobility shift assays and DNase I protection assays were carried out to confirm that a DNA probe encompassing the URE region and carrying mutations inactivating STY1 (indicated with asterisks in Figure 3A) was able to form a complex with IHF, but not with StyR-P. As well, a DNA probe encompassing the URE and carrying mutations in the IHF binding site (indicated with triangles in Figure 3A) was able to form a complex with StyR-P, but not with IHF (data not shown).

*Pa*1STY1mut and *Pa*1IHFmut were cloned in the promoter-probe vector pPR9TT, in frame with the reporter gene *lac*Z, generating pPR9STY1mut and pPR9IHFmut (Table 1; Figure 3A), respectively, and introduced in *P. fluorescens *ST. We assayed the activity of these promoters with respect to that of the wild-type promoter *Pa1*, monitoring β-galactosidase levels in inducing, repressing, and non-inducing conditions. Briefly, ST(pPR9*Pa*1), ST(pPR9STY1mut) and ST(pPR9IHFmut) strains pre-grown on styrene, were diluted and subcultured in the same medium for two hours, and then divided into three flasks containing styrene (inducing condition), styrene plus glucose (repressing condition), and glucose alone (non-inducing condition) [16,17]. The β-galactosidase levels disclosed by the different constructs along the growth in the different culture conditions are shown in Figure 3B. The styrene pre-induced strains with the different constructs diluted the accumulated β-galactosidase at the same rate during the growth on glucose, a condition in which the P*styA *promoter is not induced. This result made it possible to consider the β-galactosidase activity values obtained in this culture condition, for each strain and for each point of the growth curve, as 100% of repression of the P*styA *promoter. Similarly, we assumed the β-galactosidase values observed along the exponential growth on styrene (inducing condition) as 100% of the P*styA *activity. Since the differences in promoter activity among the different strains kept constant during the growth curve, in Figure 3C a comparison of promoter activity after two exponential cell divisions, in the different culture conditions, is reported.

**Table 1:** Bacterial strains and plasmids used in this study.

**Strains and Plasmids**	**Relevant characteristics and plasmids contruction or source**	**Reference**
**Strains**		
*P. fluorescens *ST	Sty^+^	[39]
*E. coli *DH5α	*endA1 hsdR17 supE44 thi-1 recA1 gyrA96 *(Nal^R^) *relA1*Δ (*lacIZYA-argF*)*U169 deoR*(Φ80*dlacZ*Δ*M15*)	Bethesda Res. Lab.
**Plasmids**		
pBluescript II KS+	Cloning vector; Ap^R^; 2.9 Kb.	Stratagene
pPR9TT	*lacZ *promoter probe vector; Ap^R^; Cm^R^.	[40]
pTE50	pTZ19R derivative containing a *P. fluorescens *ST chromosomal fragment carrying the genes coding for *styR*, *styA *and *styB*; Ap^R^.	[7]
pRK2013	Helper plasmid; ColE1 replicon; Mob+; Tra+; Km^R^.	[37]
pSTY1/2	155 bp fragment, encompassing nucleotides -145 to -8, with respect to the *styA *transcription start point ligated to the *Eco*RI-*Pst*I sites of pBluescript II KS+; Ap^R^.	[16]
pPR9*Pa1*	355 bp fragment, encompassing nucleotides -115 to +240, with respect to the *styA *transcription start point, ligated to the *Xho*I-*Bam*HI sites of pPR9TT; Ap^R^; Cm^R^.	[20]
pPR9STY1mut	Same construct as pPR9Pa1, but the cloned sequence carries a double mutation in positions -97 and -96 from the *styA *trascription start point; Ap^R^; Cm^R^.	This study
pPR9IHFmut	Same construct as pPR9Pa1, but the cloned sequence carries a double mutation in positions -101 and -91 from the *styA *trascription start point; Ap^R^; Cm^R^.	This study

As shown in Figures 3B and 3C, mutation of the STY1 site in pPR9STY1mut produced no effect on the promoter activity under inducing conditions with respect to pPR9*Pa*1. Thus, this StyR-P-binding site is not involved in promoter activation. On the contrary, in the contemporary presence of styrene and glucose (repressing conditions), the same mutation caused a significant relief of repression (from 60% repression disclosed by the wild-type promoter *Pa1 *to 30% repression) indicating that StyR-P bound to site STY1 plays a role in P*styA *catabolite repression. We previously demonstrated that also site STY3 is responsible for a reduction of catabolite repression from 60% to 26% [16], so it seems that these low-affinity StyR-P binding sites are both involved in P*styA *repression.

As far as IHF is concerned, we observed no β-galactosidase activity in pPR9IHFmut in all the tested culture conditions. This was an unexpected result, since in a previous work we found that the deletion of the URE region led only to a 33% reduction of promoter activity in inducing condition with respect to pPR9*Pa1 *[16]. A possible interpretation of this finding is suggested by the fact that, in spite of the different affinity of StyR-P for the single STY sites, in the absence of IHF it binds STY1 and STY2 sites simultaneously [16]. This cooperative binding suggested that two dimers of StyR-P bound to sites STY1 and STY2 could interact, bending DNA and forming a repressive loop. An indication that major changes in the three-dimensional structure of the promoter only occur when StyR-P is bound to STY1 and STY2 is the concomitant appearance of DNase I hypersensitive sites between these two sites (Figure 2, lane 1). These DNase I hypersensitive sites are not detectable when IHF displaces StyR-P from site STY1 and only STY2 is bound by StyR-P (Figure 2, lane 14). The formation of a StyR-P tetramer and DNA looping, probably favored by the presence of an intrinsically curved sequence between STY1 and STY2 [20], would impair RNA polymerase access to the promoter.

### StyR-P binding to sites STY1 and STY2 promotes protein tetramerization

In order to corroborate the hypothesis that the occupancy of both STY1 and STY2 sites by StyR-P induces the generation of a repressive DNA loop, we performed experiments aimed to assess if StyR-P dimers can form tetramers, and if this oligomerization is stimulated by the presence of a DNA fragment containing the StyR-P binding sites STY1 and STY2.

**Figure 4:**
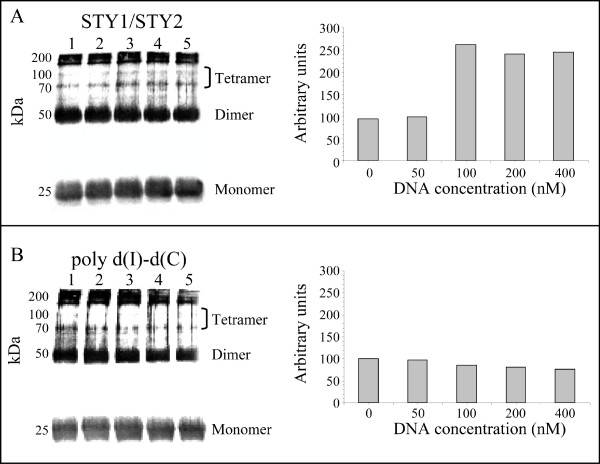
**DNA-mediated StyR-P tetramerization**. Western blot analysis of StyR-P (100 nM) incubated with increasing amounts of the DNA fragment STY1/STY2 (panel A) or poly d(I)-d(C) (panel B), and afterwards treated with the cross-linking agent DSS. Brackets indicate the 110 kDa band corresponding to StyR-P tetrameric form, and the 75 kDa band derived from incomplete cross-linking of tetramers (see text for details). Dimeric (50 kDa) and monomeric (25 kDa) StyR-P forms are indicated. The concentrations of DNA in each sample were: lane 1, no DNA added; lanes 2 to 5, 50, 100, 200, 400 nM, respectively.

To determine the oligomerization state of StyR-P, the StyR protein was phosphorylated with acetylphosphate, and afterwards treated with the disuccinimidyl suberate (DSS) cross-linking agent, in absence or in the presence of increasing amounts of a DNA fragment encompassing the STY1 and STY2 sites. As shown in Figure 4A, StyR-P can form dimers of the predicted size of about 50 kDa and higher order multimers. The size of the higher-order multimers of about 75 kDa and 110 kDa could be consistent with the formation of trimers and tetramers, respectively. However, native PAGE analysis performed on the acetylphosphate-treated sample showed that more than 99% of StyR was in the dimeric form, prior to the addition of DSS (data not shown) [12]. After cross-linking and denaturing SDS-PAGE analysis, more than 50% of the protein migrated as the monomeric form. This indicates that the extent of protein-protein cross-linking was far from complete, under these experimental conditions. Thus, it is likely that the band of about 75 kDa derives from incomplete cross-linking of a tetramer rather than from the formation of a trimer.

The results of the cross-linking experiment highlight that StyR-P dimers are actually able to interact, forming a tetramer, also in the absence of DNA. Tetramers formation is significantly enhanced by addition of a DNA fragment encompassing sites STY1 and STY2 (Figure 4A), but not by the addition of non-specific DNA (Figure 4B). This specific DNA-mediated oligomerization is a strong indication that two StyR-P dimers bound on sites STY1 and STY2 form a StyR-P tetramer, likely inducing the formation of a DNA loop. Occurrence of changes in the promoter conformation when StyR-P is bound to both STY1 and STY2 is also indicated by the appearance of hypersensitive bands between these sites in DNase I protection experiments (Figure 2). Moreover, a strong evidence of the formation of a "closed" promoter conformation that would impair RNA polymerase (RNAP) access to the promoter is the absence of P*styA *activity in pPR9IHFmut. Taken together the above evidence concur to strengthen the significance of the cross-linking results.

### IHF has a positive role on PstyA activity by itself

**Figure 5:**
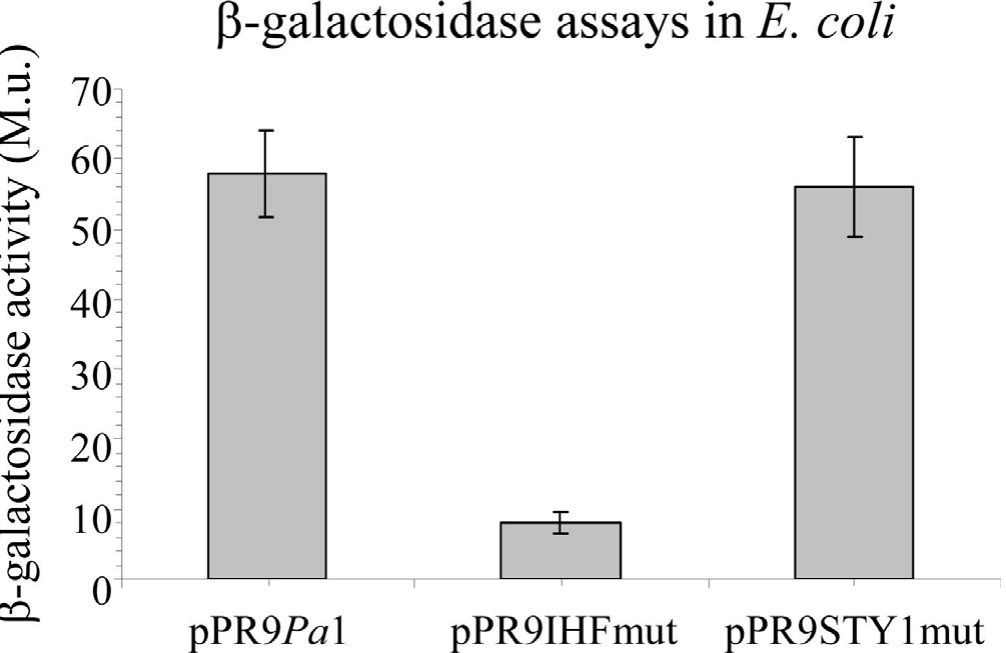
**Role of IHF on P*styA *activity**. β-galactosidase activities disclosed by *E. coli *strains carrying the pPR9TT-derivative plasmids indicated below the histogram. The β-galactosidase assays were carried out on cells grown in LB medium at 37°C to an A_600 _≅ 2.0. Standard deviations (vertical lines) are based on the mean values of five independent experiments.

The above reported molecular and physiological results concur with the view that the cooperative binding of StyR-P to STY1 and STY2 is likely counteracted *in vivo *by IHF which, competing with StyR-P for binding to the URE region, would act as a positive modulator of promoter activity. However, in a previous work we found that deletion of the entire URE led to a 33% decrease in promoter activity in inducing conditions (growth on styrene as sole carbon source) with respect to the full length promoter [16]. From this result we argued that IHF could not merely have the role of displacing StyR-P from the repressive site STY1, since in this case no difference in promoter activity would have been found between the full length and the URE-deleted promoters. To clarify this issue, we compared the activity of pPR9*Pa*1 and pPR9IHFmut in the heterologous host *E. coli*. As a control we also tested the β-galactosidase activity of pPR9STY1mut (Figure 5). In this system there was no StyR-P dependent activation of P*styA*, but the promoter had a basal level activity anyway. This basal level activity was the same in pPR9*Pa*1 and in pPR9STY1mut, while it strongly decreased in pPR9IHFmut. This result demonstrates that IHF, beside displacing StyR-P from the STY1 repressive site, has also a positive effect on P*styA *transcription by itself.

The molecular mechanism of action of IHF on the P*styA *promoter remains an open issue. In many cases IHF binding induces DNA-looping facilitating the interaction of an upstream regulatory element with RNAP [21]. However, our data rule out this possibility, since the IHF binding site is located at the very 5'- end of the minimum P*styA *fragment endowed with full activity [20]. Therefore, it is likely that IHF acts as an activator through a different mechanism. For instance, it has been reported that IHF can activate transcription, without contacting RNAP, by facilitating duplex destabilization in the -10 region [22,23].

### P*styA *catabolite repression does not rely on variation in StyR and IHF protein levels

Although the role of IHF in *Pseudomonas *physiology is far from understood, it has been often involved in the regulation of toxic compound degradation [18]. The above data show that binding of IHF to the URE exerts a positive effect on P*styA *activity, under inducing conditions, while binding of StyR-P on the same region is involved in catabolite repression. Since StyR-P and IHF are in binding competition for the URE region, it is reasonable to suppose that, starting from an inducing condition (growth on styrene as sole carbon source), the glucose-mediated repression (growth on styrene and glucose) of P*styA *could be due to an increase in StyR-P and/or to a decrease in IHF cellular levels.

To test this hypothesis, we monitored the levels of StyR and IHF proteins by western analysis after the addition of glucose to a styrene growing culture.

**Figure 6:**
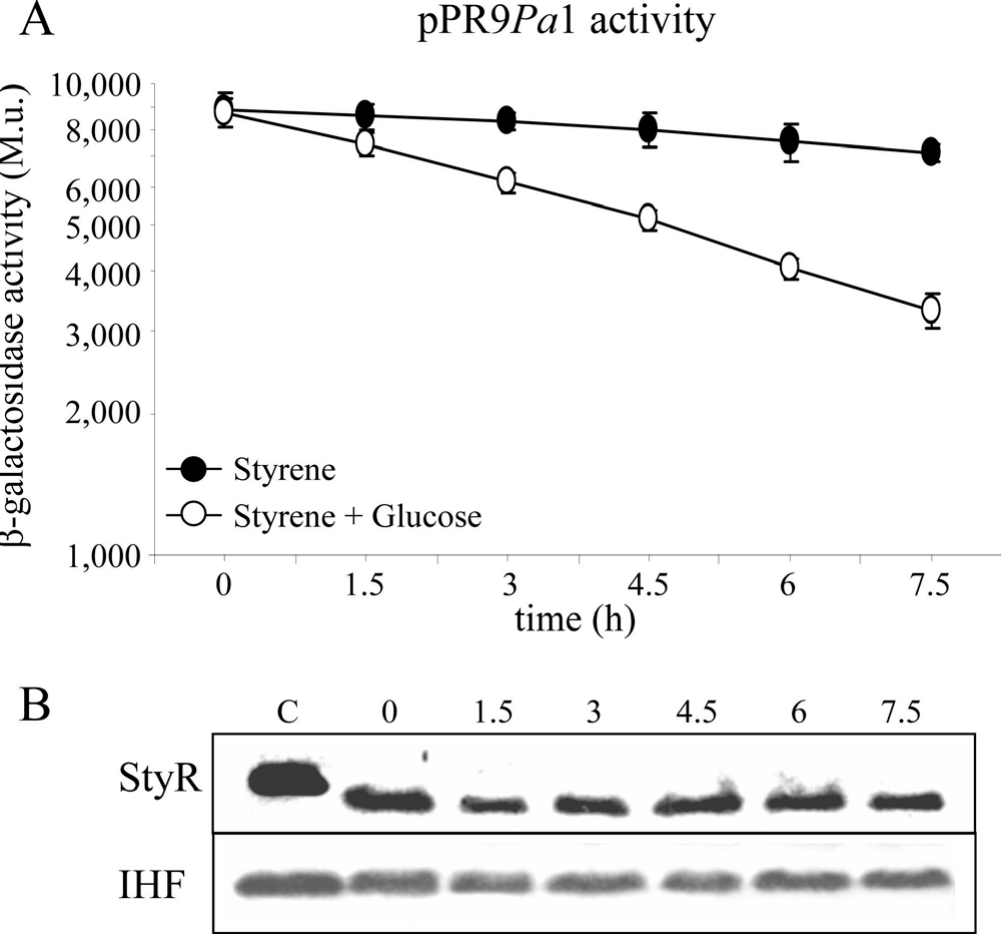
**Analysis of StyR and IHF protein levels**. (A) β-galactosidase assays performed with *P. fluorescens *ST strains carrying the pPR9*Pa*1 plasmid (Table 1), grown in styrene (filled circles) and styrene plus 0.4% (wt/vol) glucose (open circles), along the growth curve. (B) Western hybridization performed with anti-StyR and anti-IHF antibodies on the cellular soluble fractions derived from the styrene plus glucose culture. Sampling times are indicated above the bands, and correspond to the sampling times of the β-galactosidase assays shown in (A); lane C, control with purified StyR and IHF proteins.

As previously described, *P. fluorescens *ST carrying pPR9*Pa*1 plasmid was cultured with styrene as sole carbon source, then the culture was diluted, subcultured in the same medium for two hours, and divided into two flasks containing styrene, or styrene plus glucose. β-galactosidase levels were measured every 1.5 hours and protein samples for western analysis were withdrawn, in parallel, up to 7.5 hours after division. In inducing condition, StyR and IHF levels did not vary along the growth curve (data not shown), in accordance with P*styA *steady levels (Figure 6A). Surprisingly, IHF levels did not vary and StyR levels did not increase, even when glucose was added to the styrene growing culture (Figure 6B), although P*styA *activity decreased in this culture condition (Figure 6A).

This finding strongly suggests that in repressing conditions the low-affinity site STY1 is occupied as a consequence of an increase in the levels of phosphorylated StyR, and not for an increase in StyR or for a decrease in IHF absolute protein levels.

## Conclusion

In our previous studies we showed that StyR dimerizes upon phosphorylation and that StyR-P dimers are able to bind P*styA *on three distinct *cis*-acting elements (STY1, STY2 and STY3) with different affinity [15,16]. Functional studies showed that the high-affinity binding site STY2 is essential for promoter activation, while the low-affinity site STY3 is in part responsible for catabolite repression of the promoter. The URE region, encompassing the StyR-P binding site STY1 and the IHF binding site, is involved in both styrene-induction and catabolite repression of P*styA *[16].

In this study we unravel the role of the URE region in P*styA *regulation, showing that the mutually exclusive binding of StyR-P and IHF to this *cis*-acting element causes opposite effects on P*styA *activity. Moreover, we demonstrate that the levels of IHF and StyR do not change in the different growth conditions, making the phosphorylation degree of StyR the main factor in the regulation of styrene catabolism genes expression.

**Figure 7:**
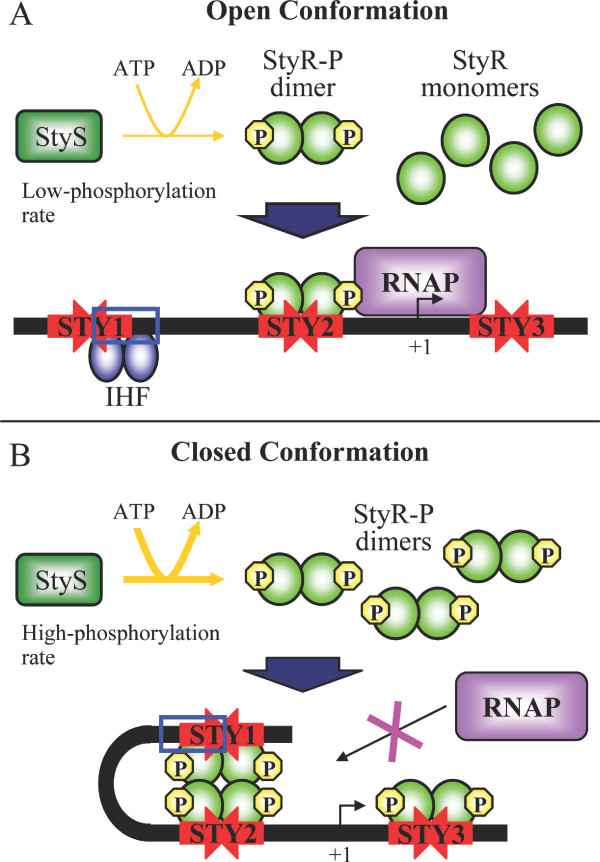
**Proposed model for the regulation of P*styA***. (A) When growing on styrene as sole carbon source, the StyS kinase activity leads to levels of StyR-P such that this protein binds only to the high-affinity actvating-site STY2, while IHF binds to the URE region. This "open" conformation would allow the binding of the RNA polymerase (RNAP) to P*styA*, promoting transcription. (B) In carbon catabolite repression conditions (growth on styrene plus glucose) the increased StyS kinase activity would lead to higher levels of StyR-P, so that this protein can bind also to the low-affinity repressive-sites STY1 and STY3, displacing IHF from the URE region. This "closed" promoter conformation would impair the binding of the RNAP to the promoter. The balance between open and closed promoter conformations would determine a fine modulation of the promoter activity.

On the whole, our research allows us to propose a model for P*styA *regulation according to which the P*styA *activity is determined by changes of its structure. An open conformation is operative in inducing conditions when StyR-P is bound to STY2 site and IHF to the URE. Under catabolite repression conditions StyR-P cellular levels would increase, displacing IHF from the URE and resulting in the achievement of a promoter closed conformation. Also the occupation of STY3 site would take part to the closed promoter conformation, since we had previously demonstrated that its inactivation partially relieved P*styA *from glucose repression (Figure 7) [16]. The balance between the open and the closed promoter conformations would determine a fine modulation of P*styA *activity.

In this perspective the key-factor regulating carbon catabolite repression would be the activity of the StyR-cognate sensor protein StyS. Our data converge to the notion that the activity of the sensor kinase StyS is enhanced when, besides styrene, cells are in a condition that determines a high redox potential (such as growth on styrene plus glucose). This view is consistent with the presence of two PAS domains and two kinase domains in the StyS sensor, which is a strong indication that two different signals can be sensed by this protein. If the two signals are styrene and the cell redox potential, a signal often perceived by PAS domains, their integration could modulate the kinase activity of the sensor, leading to an increase in the levels of phosphorylated StyR, under carbon catabolite repression conditions, and consequently to P*styA *down-regulation [24].

Concerning the physiological role of IHF, in *E. coli *this protein regulates more than 100 genes and in *Pseudomonas *it is involved in the regulation of several processes, especially in toxic compound degradation [18,25]. Previous studies showed that in *E. coli *and *Pseudomonas *IHF levels increase upon entry into stationary phase in LB growing cultures and suggested a role for IHF in stress response [26,27]. It is likely that in *P. fluorescens *ST we did not observe the same phenomenon because we used minimal medium or because styrene is a stress factor by itself.

The TodS/T regulatory system of toluene catabolism in *P. putida *strain DOT-T1E strongly resembles the StyS/R system. The TodT response regulator binds with different affinity three sites of the promoter P*todX *and IHF is essential for activating transcription [28,29]. However, in this system all these TodT binding sites have a positive effect on promoter activation and the role of IHF is to bend DNA, hence favouring the contact between the TodT activator bound further upstream and the α-subunit of RNA polymerase. Accordingly, the position of TodT and IHF binding sites on P*todX *promoter is very different from that of StyR and IHF binding sites on P*styA*. The divergent evolution of StyS/R-*PstyA *and TodS/T-P*todX *probably reflects the need for integrating these peripheral pathways into the global regulatory circuits of the corresponding different strains.

In *Pseudomonas *the majority of the catabolic routes for toxic compounds are regulated by a specific regulator responding to the actual substrate and by one or more global regulators devoted to couple the activity of the catabolic operon promoter to cell metabolism [18,19,30]. With this respect, the styrene-catabolism regulatory system is peculiar because a unique regulatory device (the StyS/StyR two component system) integrates the response to the specific stimulus (styrene) with the utilization of a higher-energy carbon source.

The fine modulation exerted by the interplay of StyR-P and IHF on P*styA *activity, although complex, could not be the only regulatory device controlling styrene catabolism in *P. fluorescens *ST. It was recently reported that in *Pseudomonas *sp. Y2, in which the *sty *regulatory and catabolic genes are highly homologous to those of strain ST, the repressor PaaX represses P*styA *in the absence of phenylacetyl-CoA, the first intermediate metabolite of the styrene degradation lower-pathway [31]. The authors suggested that PaaX could have a role in coordinating the expression of the upper and lower styrene catabolic pathways to avoid the accumulation of toxic catabolic intermediates such as styrene oxide and phenylacetaldehyde. We believe that both StyR-P and PaaX repressive mechanisms can be operative also in ST strain, probably acting in different metabolic conditions.

In the experimental design here reported we found that when styrene-induced cells are transferred to a medium containing both styrene and glucose, both carbon sources are simultaneously utilized (since P*styA *activity is diminished, but not abrogated). Conversely, when non-induced cells are transferred to a medium containing both glucose and styrene, they exhibit a typical diauxic growth, in which glucose is utilized first [17]. Therefore it seems that catabolite repression could be achieved through different mechanisms, depending on the metabolic status of the cells preceding the addition of the alternative carbon source. It could be that when styrene is added to a glucose growing culture, the available energy is invested in adaptation to the high-toxic styrene, prior to metabolizing it. Conversely, when cells are already adapted to styrene and are productively using it as carbon source, there is not much advantage to completely repress styrene degradation in favor of glucose utilization, and the two carbon sources are thus exploited in parallel. The simultaneous utilization of an high-energy carbon source and of an aromatic compound has also been reported by J.L. Ramos and co-workers, who demonstrated that in *P*. putida KT2440 (pWWO) cells utilize glucose and toluene simultaneously [32].

In addition to its relevance in understanding the physiological mechanisms underlying aromatic compound catabolism, this study could lay the basis for the generation of engineered strains desensitized to carbon catabolite repression, thus improved in styrene catabolic potential.

## Methods

### Bacterial strains, plasmids, media and chemicals

The bacterial strains and plasmids used in this study are listed in Table 1. *P. fluorescens *ST and *E. coli *cells were routinely grown at 30°C and 37°C, respectively, in Luria-Bertani (LB) medium or mineral salts medium supplemented with styrene, or 0.4% (wt/vol) glucose, or both [33,34]. Styrene was added via the gas phase as previously described [7]. When necessary, cultures were supplemented with ampicillin (Ap, 100 μg/ml), kanamycin (Km, 50 μg/ml) or chloramphenicol (Cm, 30 μg/ml for *E. coli*; 200 μg/ml for *P. fluorescens *ST).

### Recombinant DNA techniques

Details on the construction of plasmids are described in Table 1. Preparation of plasmid DNA, purification of DNA fragments, restrictions, ligations and transformations of *E. coli*, were carried out by standard procedures [15,33]. PCR amplifications were performed using *Pfu *polymerase (Stratagene) and the pTE50 plasmid as DNA template (Table 1) [7]. The sequences of oligonucleotides used in this study are shown in Table 2. Automated sequences were performed by MWG Biotech sequence services (MWG Biotech).

**Table 2:** Oligonucleotides used in this study.

**Name**	**Sequence (5'-3')^a^**	**Position^b^**	**Site^c^**
P1/FW	GCTCTAGAGGTGTAGTAAATATAAGT	-115	*Xba*I
P169/FW	GCTCTAGAGGTGTAGTAAATATTAGT	-115	*Xba*I
P87/FW	TAAATATAAGT**AA**ATGATTTTTAAT	-108	*-*
P89/FW	TAAATAT**T**AGTTTATGA**A**TTTTAAT	-108	-
P86/RV	ATTAAAAATCAT**TT**ACTTATATTTA	-84	*-*
P88/RV	ATTAAAA**T**TCATAAACT**A**ATATTTA	-84	*-*
P17/RV	GGGGTACCTACGTAGTAGTAGTGG	+274	*Kpn*I
P35/FW	GGGAATTCCGTTGACTGCTTCGGG	-145	*Eco*RI
P56/RV	AAACTGCAGAGCTAACACCAGCAGC	-8	*Pst*I

^a ^introduced restriction sites are underlined; the nucleotides in bold-face and underlined correspond to the A → T and T → A introduced substitutions with respect to the *styA *wild-type sequence.

^b ^distance in bp from P*styA *transcription start point [10].

^c ^restriction recognition sites.

### DNase I protection assay

The DNase I protection assay was performed as previously described [16]. In brief, the probe derived from *Eco*RI/*Sac*I digestion of the pSTY1/2 plasmid (Table 1) and encompassing nucleotides -145/-8 with respect to P*styA *transcription start point was labelled by fill-in with [α-^32^P] dATP [16]. The labelled probe (0.5 nM concentration) was mixed with different amounts of phosphorylated StyR (StyR-P) (0.5 to 8.0 μM, as indicated in legend of Figure 2) and/or IHF (0.5 to 8.0 μM, as indicated in legend of Figure 2) in "Phosphorylation Buffer" (43 mM Tris-acetate pH 8.0, 30 mM potassium acetate, 8 mM MgCl_2_, 27 mM ammonium acetate, 1 mM DTT, 80 mM KCl, 10% (vol/vol) glycerol, 4% (wt/vol) polyethylene glycol, 100 μg/ml bovine serum albumin) containing 0.1 μg/μl poly(dI-dC) and 2 mM CaCl_2_. *In vitro *phosphorylation of StyR was previously described [15]. DNA-protein complexes were allowed to form at 30°C for 15 min in a total volume of 50 μl for reaction. After 1 min at 25°C, DNase I (0.4 u; Roche Biochemicals) was added to the reaction mixtures. The reaction mixture was incubated for 1 min at 25°C and then stopped by the addition of 150 μl of "Stop Solution" (0.2 M sodium acetate pH 7.0, 0.1 M ethylenediaminetetraacetic acid pH 8.0, 0.15% (wt/vol) sodium dodecyl sulfate, 100 μg/ml tRNA). DNA from the footprinting mixture was phenol-chloroform extracted, ethanol-precipitated and dissolved in 5 μl of "Sequence Loading Buffer" [33]. After 3 min denaturation at 95°C, DNA was loaded on a 7% DNA sequencing gel [33]. The A+G Maxam and Gilbert reaction was carried out with the same probe and loaded on the gel along with the footprinting samples [33].

### Construction of P*styA::lacZ *fusions and β-galactosidase assays

The construction of pPR9TT-derivatives pPR9*Pa*1 has been previously described [20]. For the construction of plasmids pPR9STY1mut and pPR9IHFmut, a site-directed mutagenesis of P*styA *was performed by the "Splicing by Overlap Extension PCR" method using the primers described in Table 2, as previously described [35,36]. These oligonucleotides are mutually complementary and correspond to a DNA region located from nt -108 to -84 with respect to the *styA *transcription start point. The A → T and T → A introduced substitutions, with respect to the wild-type sequence, are indicated in Table 2. In the first step, two distinct PCR reactions (PCR-1 and PCR-2) were carried out using chromosomal plasmid pTE50 (Table 1) as template to introduce the mutation in the PCR products. In the PCR-1 *Pa*1STY1mut was amplified with primers P1/FW and P86/RV, while *Pa*1IHFmut with primers P169/FW and P88/RV. In the PCR-2 reactions, the reverse primer was P17/RV, while the forward primer was P87/FW for *Pa*1STY1mut and P89/FW for *Pa*1IHFmut. In the second PCR step the products from PCR-1 and PCR-2 were used as both primers and templates. In this step a total DNA amount (PCR-1 + PCR-2) of 125 ng has been used in a 100 μl reaction. After the first 5 cycles (94°C, 1 min; 68°C, 1 min; 72°C, 1 min), the reverse primer P17/RV and either primer: P1/FW or P169/FW, were added and the reaction was continued for 25 cycles.

The different PCR products were first blunt-cloned in *Hinc*II-digested pBluescriptII KS+ (Stratagene) and checked by sequencing. Afterwards, the fragments cloned in the right orientation were excised by *Xho*I-*Bam*HI digestion and ligated to compatible sites of the promoter probe vector pPR9TT (Table 1) in frame with the *lacZ *reporter gene. All pPR9TT-derivatives were transferred from *E. coli *to *P. fluorescens *ST by triparental matings with the helper plasmid pRK2013 (Table 1) [37].

In order to measure β-galactosidase activity, *P. fluorescens *ST cells harboring pPR9TT-derived plasmids, were grown for 12 h at 30°C in mineral salts medium (MM) supplemented with styrene as carbon source; cells were then diluted to a cell density corresponding to A_600 _≅ 0.05 in the same medium and subcultured until cultures reached A_600 _≅ 0.2. Cultures were then divided into three flasks and 0.4% (wt/vol) glucose was added to one flask. Samples were withdrawn, every 1.5 hours, during the entire growth curve and β-galactosidase activity was measured as described by Miller [38]. The β-galactosidase assays performed in the *E. coli *strains carrying the different pPR9TT-derivative plasmids were carried out on cells grown in LB medium at 37°C to an A_600 _≅ 2.0.

### Protein-protein cross-linking assay

The DNA fragment STY1/STY2 (nucleotides -145 to -8 with respect to P*sty*A transcription start point) used to promote StyR-P tetramerization was obtained by PCR amplification with the primers P35/FW and P56/RV described in Table 2. StyR-P (100 nM) was incubated with different concentrations of the STY1/STY2 PCR product or in presence of poly d(I)-d(C) (from 50 to 400 nM) in "Binding Buffer" (20 mM Tris-HCl, 2 mM EDTA, 5 mM MgCl_2_, 30 mM KCl, 5% (vol/vol) glycerol, 0.025% (vol/vol) Nonidet P-40, and 30 μg/ml poly (dI-dC); pH 8.0). After 15 min of incubation at 30°C, disuccinimidyl suberate (DSS) was added to a final concentration of 15 mM, followed by incubation at room temperature for 60 min. To stop the cross-linking reaction, 5 μl of "6X Protein Sample Buffer" was added to the samples [33]. Cross-linking products were separated by electrophoresis on a 10% (wt/vol) sodium dodecyl sulfate-polyacrylamide gel [33]. Proteins were electroblotted to nitrocellulose and StyR was detected with murine anti-6xHis monoclonal antibodies (Qiagen) and alkaline phosphatase-conjugate goat anti-mouse secondary antibody [15].

### Polyclonal antiserum production and Western analyses

Purified StyR (200 μg) was emulsioned with complete Freund's adjuvant (Sigma) and used to immunize a mouse by intramuscular injection. After 3 weeks, a second boost of StyR (150 μg) in complete Freund's adjuvant (Sigma) was given, followed by a third boost (100 μg) at the sixth week. The animal was bled 2 weeks later and the serum was stored at 4°C. Animal experiments were performed according to the legislative decree 116/92 by the Italian Ministry of Health. Western blotting and immunohybridization were carried out with anti-StyR antiserum (1:1,500), anti-IHF antiserum (1:1,000) and alkaline phosphatase-conjugate anti-mouse IgG as secondary antibody (1:5,000; Promega) [33]. Final development was performed with the "BCIP/NBT Color Development Substrate" (Promega), as recommended by the manifacturer. Anti-IHF antiserum was a generous gift from Prof. S.D. Goodman, University of Southern California, Los Angeles, CA, USA.

## Authors' contributions

GR and LL performed research, participated in the design of the study and helped to write the manuscript. BP participated in the design of the study. EZ conceived of the study, participated in its design and coordination, and wrote the manuscript. All authors read and approved the final manuscript.

## References

[B1] U.S. Environmental Protection Agency, Integrated Risk Information System, Styrene. http://www.epa.gov/iris/subst/0104.htm.

[B2] O'Leary ND, O'Connor KE, Dobson ADW (2002). Biochemistry, genetics and physiology of microbial styrene degradation. FEMS Microbiol Rev.

[B3] Otto K, Hofstetter K, Röthlisberger M, Witholt B, Schmid A (2004). Biochemical characterization of StyAB from *Pseudomonas *sp. Strain VLB120 as a two-component flavin-diffusible monooxygenase. J Bacteriol.

[B4] Kantz A, Chin F, Nallamothu N, Nguyen T, Gassner GT (2005). Mechanism of flavin transfer and oxygen activation by the two-component flavoenzyme styrene monooxygenase. Arch Biochem Biophys.

[B5] Utkin IB, Yakimov MM, Matveeva LN, Kozlyak EI, Rogozhin IS, Solomon ZG, Bezborodov AM (1991). Degradation of styrene and ethylbenzene by *Pseudomonas *species Y2. FEMS Bicrobiol Lett.

[B6] O'Connor K, Buckley CM, Hartmans S, Dobson ADW (1995). Possible regulatory role for nonaromatic carbon sources in styrene degradation by *Pseudomonas putida *CA-3. Appl Environ Microbiol.

[B7] Marconi AM, Beltrametti F, Bestetti G, Solinas F, Ruzzi M, Galli E, Zennaro E (1996). Cloning and characterization of styrene catabolism genes from *Pseudomonas fluorescens *ST. Appl Environ Microbiol.

[B8] Beltrametti F, Marconi AM, Bestetti G, Colombo C, Galli E, Ruzzi M, Zennaro E (1997). Sequencing and functional analysis of styrene catabolism genes from *Pseudomonas fluorescens *ST. Appl Environ Microbiol.

[B9] Panke S, Witholt B, Schmid A, Wubbolts MG (1998). Towards a biocatalyst for (*S*)-styrene oxide production: characterization of the styrene degradation pathway of *Pseudomonas *sp. Strain VLB120. Appl Environ Microbiol.

[B10] Velasco A, Alonso S, Garcìa JL, Perera J, Dìaz E (1998). Genetic and fuctional analysis of the styrene catabolic cluster of *Pseudomonas *sp. Strain Y2. J Bacteriol.

[B11] Mooney A, O'Leary ND, Dobson ADW (2006). Cloning and functional characterization of the *styE *gene, involved in styrene transport in *Pseudomonas putida *CA-3. Appl Environ Microbiol.

[B12] Leoni L, Rampioni G, Zennaro E, Ramos JL, Filloux A (2007). Styrene, an unpalatable substrate with complex regulatory networks. Pseudomonas, a model system in biology.

[B13] Luengo JM, Garcia JL, Olivera EL (2001). The phenylacetyl-CoA catabolon: a complex catabolic unit with broad biotechnological applications. Mol Microbiol.

[B14] Milani M, Leoni L, Rampioni G, Zennaro E, Ascenzi P, Bolognesi M (2005). An active-like structure in the unphosphorylated StyR response regulator suggests a phosphorylation-dependent allosteric activation mechanism. Structure.

[B15] Leoni L, Ascenzi P, Bocedi A, Rampioni G, Castellini L, Zennaro E (2003). Styrene-catabolism regulation in *Pseudomonas fluorescens *ST: phosphorylation of StyR induces dimerization and cooperative DNA-binding. Biochem Biophys Res Comm.

[B16] Leoni L, Rampioni G, Di Stefano V, Zennaro E (2005). Dual role of response regulator StyR in styrene catabolism regulation. Appl Environ Microbiol.

[B17] Santos PM, Blatny JM, Di Bartolo I, Valla S, Zennaro E (2000). Physiological analysis of expression of styrene degradation cluster in *Pseudomonas fluorescens *ST. Appl Environ Microbiol.

[B18] Shingler V (2003). Integrated regulation in response to aromatic compounds: from signal sensing to attractive behaviour. Environ Microbiol.

[B19] Cases I, de Lorenzo V (2005). Promoters in the environment: transcriptional regulation in its natural context. Nat Rev Microbiol.

[B20] Santos PM, Leoni L, Di Bartolo I, Zennaro E (2002). Integration host factor is essential for the optimal expression of *styABCD *operon in *Pseudomonas fluorescens *ST. Res Microbiol.

[B21] McLeod SM, Johnson RC (2001). Control of transcription by nucleoid proteins. Curr Opin Microbiol.

[B22] Sheridan SD, Benham CJ, Hatfield GW (1998). Activation of gene expression by a novel DNA structural transmission mechanism that requires supercoiling-induced DNA duplex destabilization in an upstream activating sequence. J Biol Chem.

[B23] Sze CC, Laurie AD, Shingler V (2001). *In vivo *and *in vitro *effects of integration host factor at the DmpR-regulated sigma(54)-dependent Po promoter. J Bacteriol.

[B24] Taylor BL, Zhulin IB (1999). PAS domains: internal sensors of oxygen, redox potential and light. Microbiol Mol Biol Rev.

[B25] Arfin SM, Long AD, Ito ET, Tolleri L, Riehle MM, Paegle ES, Hatfield GW (2000). Global gene expression profiling in *Escherichia coli *K12. J Biol Chem.

[B26] Aviv M, Giladi H, Schreiber G, Oppenheim AB, Glaser G (1994). Expression of the genes coding for the *Escherichia coli *integration host factor are controlled by growth phase, *rpoS*, ppGpp and by autoregulation. Mol Microbiol.

[B27] Valls M, Buckle M, de Lorenzo V (2001). *In vivo *UV laser footprinting of the *Pseudomonas putida *sigma 54 Pu promoter reveals that integration host factor couples transcriptional activity to growth phase. J Biol Chem.

[B28] Lacal J, Busch A, Guazzaroni ME, Krell T, Ramos JL (2006). The TodS-TodT two-component regulatory system recognizes a wide range of effectors and works with DNA-bending proteins. Proc Natl Acad Sci USA.

[B29] Lacal J, Guazzaroni ME, Busch A, Krell T, Ramos JL (2008). Hierarchical binding of the TodT response regulator to its multiple recognition sites at the *tod *pathway operon promoter. J Mol Biol.

[B30] Cases I, de Lorenzo V (2001). The black cat/white cat principle of signal integration in bacterial promoters. EMBO J.

[B31] del Peso-Santos T, Bartolomé-Martín D, Fernández C, Alonso S, García JL, Díaz E, Shingler V, Perera J (2006). Coregulation by phenylacetyl-coenzyme A-responsive PaaX integrates control of the upper and lower pathways for catabolism of styrene by *Pseudomonas *sp. strain Y2. J Bacteriol.

[B32] del Castillo T, Ramos JL (2007). Simultaneous catabolite repression between glucose and toluene metabolism in *Pseudomonas putida *is channeled through different signaling pathways. J Bacteriol.

[B33] Sambrook J, Fritsch EF, Maniatis T (1989). Molecular cloning: a laboratory manual.

[B34] Hartmans S, Smits JP, Werf MJ van der, Volkering F, de Bont JA (1989). Metabolism of styrene oxide and 2-phenylethanol in the styrene-degrading *Xanthobacter *strain 124X. Appl Environ Microbiol.

[B35] Horton RM, Hunt HD, Ho SN, Pullen JK, Pease LR (1989). Engineering hybrid genes without the use of restriction enzymes: gene splicing by overlap extension. Gene.

[B36] Rampioni G, Polticelli F, Bertani I, Righetti K, Venturi V, Zennaro E, Leoni L (2006). The *Pseudomonas *quorum-sensing regulator RsaL belongs to the tetrahelical superclass of H-T-H proteins. J Bacteriol.

[B37] Figursky DH, Helinski DR (1979). Replication of an origin-containing derivative of plasmid RK2 dependent on a plasmid function provided in *trans*. Proc Natl Acad Sci USA.

[B38] Miller JH (1972). Experiments in in molecular genetics.

[B39] Baggi G, Boga MM, Catelani E, Galli E, Treccani V (1983). Styrene catabolism by a strain of *Pseudomonas fluorescens*. Syst Appl Microbiol.

[B40] Santos PM, Di Bartolo I, Blatny JM, Zennaro E, Valla S (2001). New broad-host-range promoter probe vectors based on the plasmid RK2 replicon. FEMS Microbiol Lett.

